# Optimization of Ultrasound-Assisted Extraction of Antioxidants from the Mung Bean Coat

**DOI:** 10.3390/molecules22040638

**Published:** 2017-04-15

**Authors:** Yue Zhou, Jie Zheng, Ren-You Gan, Tong Zhou, Dong-Ping Xu, Hua-Bin Li

**Affiliations:** 1 Guangdong Provincial Key Laboratory of Food, Nutrition and Health, School of Public Health, Sun Yat-Sen University, Guangzhou 510080, China; zhouyue3@mail2.sysu.edu.cn (Y.Z.); zhengj37@mail2.sysu.edu.cn (J.Z.); zhout43@mail2.sysu.edu.cn (T.Z.); xudp@mail2.sysu.edu.cn (D.-P.X.); 2 School of Biological Sciences, The University of Hong Kong, Hong Kong 999077, China; ganry@connect.hku.hk; 3 South China Sea Bioresource Exploitation and Utilization Collaborative Innovation Center, Sun Yat-Sen University, Guangzhou 510006, China

**Keywords:** mung bean coat, antioxidant, ultrasound-assisted extraction, optimization, response surface methodology, waste, value-added utilization

## Abstract

Mung bean (*Vigna radiata*) sprout is commonly consumed as a vegetable, while the coat of the germinated mung bean is a waste. In this paper, an ultrasound-assisted extraction method has been developed to extract natural antioxidants from the seed coat of mung bean. Several experimental parameters—which included ethanol concentration, solvent/material ratio, ultrasound extraction time, temperature, and power—were studied in single-factor experiments. The interaction of three key experimental parameters (ethanol concentration, solvent/material ratio, and ultrasonic extraction time) was further investigated by response surface method. Besides, traditional extracting methods, including maceration and Soxhlet extraction methods, were also carried out for comparison. The results suggested that the best extracting condition was 37.6% (*v*/*v*) of ethanol concentration, 35.1:1 mL/g of solvent/material ratio and ultrasonic extraction of 46.1 min at 70 °C under 500 W ultrasonic irradiation. The antioxidant capacity (178.28 ± 7.39 µmol Trolox/g DW) was much stronger than those obtained by the maceration extraction process (158.66 ± 4.73 µmol Trolox/g DW) and the Soxhlet extraction process (138.42 ± 3.63 µmol Trolox/g DW). In addition, several antioxidant components in the extract were identified and quantified. This study is helpful for value-added utilization of the waste from germinated mung bean.

## 1. Introduction

The excessive free radicals play a key role in a large number of diseases because they cause damage to DNA, lipids, and proteins. Natural antioxidants are regarded as potential pharmaceuticals for oxidative stress-induced diseases [1]. Natural antioxidants exist widely in edible plants including fruits, vegetables, cereals, flowers, herbs, and legumes [2,3,4,5,6,7,8,9]. Natural antioxidants have attracted worldwide attention because of their application in food, cosmetic, and pharmaceutical industries, so development of efficient extraction methods of antioxidants are needed.

Mung bean (*Vigna radiata* L.) is one of the most popular dietary legumes in Asian countries, especially in India and China. It contains a lot of protein, and its processed products are also rich in nutrients [10]. Mung bean sprout is also widely consumed by Asian people as a green vegetable, and it is rich in fibers, vitamins, and polyphenols, which contribute to the biological activities of mung bean. According to research, there are more significant bioactivities and more secondary metabolites in the sprouts of mung beans because some biosynthetic enzymes are activated during the germination process [11,12]. The vitamins and polyphenols (the flavonoids isovitexin and vitexin) are mainly present in the mung bean seed coats [10,13,14]. However, the germinated mung bean coat (GMBC) is usually abandoned as waste before being consumed. If the antioxidants of GMBC were extracted effectively, they could be applied as a new source of plant-derived antioxidants.

Extraction is essential during the process of separation and identification of compounds from solid samples. Many extraction methods have been used to extract natural antioxidants from solid samples, such as maceration extraction, Soxhlet extraction, microwave-assisted extraction, ultrasound-assisted extraction (UAE), and supercritical fluid extraction. UAE is one of the most effective extraction methods, and it is a simple, rapid, and low-cost method [15,16]. UAE method has been used to extract several antioxidants from plant matrix [17,18,19,20]. Ultrasound technique is employed to extract compounds from plants because of its high frequency ultrasonic waves. The waves induce contraction and expansion cycles and cause cavitation, thus breaking the cell walls of plants and assisting the infiltration of the solvent [17]. The extraction rate and yield of UAE are influenced by a number of factors, including solvent concentration; solvent/material ratio; ultrasonication time, temperature, power; etc.

Response surface method (RSM) is a mathematical tool which can be used to obtain optimal parameters with the least experiments. It evaluates individual and interactive influences of different factors and also predicts the outcome of variables under the predefined condition [15]. UAE of antioxidants from GMBC with surface response method has not been reported in the literature. In this paper, the main purpose is to optimize extraction of antioxidant ingredients from GMBC, and different concentrations of ethanol; solvent/material ratios; ultrasonication times, temperatures, and powers were evaluated in the single-factor experiments. The interaction of three key experimental parameters was studied using response surface method with central composite rotatable design. The main antioxidant components in the extract obtained under the optimized extraction conditions were identified and quantified using high-performance liquid chromatography. Besides, Soxhlet and maceration extraction methods were also performed for comparison.

## 2. Results and Discussion

### 2.1. Single-Factor Experiments

Single-factor experiments were carried out to evaluate the effect of every factor on yield of antioxidant ingredients in the GMBC extracts. In this section, effects of several important factors were investigated: ethanol concentration (10%–60%), the solvent/material (S/M) ratio (10:1–60:1 mL/g), ultrasonication time (0–75 min), temperature (40–90 °C) and power (300–800 W). Major influence factors obtained in the single-factor experiments were applied in the response surface method design.

#### 2.1.1. Ethanol Concentration

**Figure 1:**
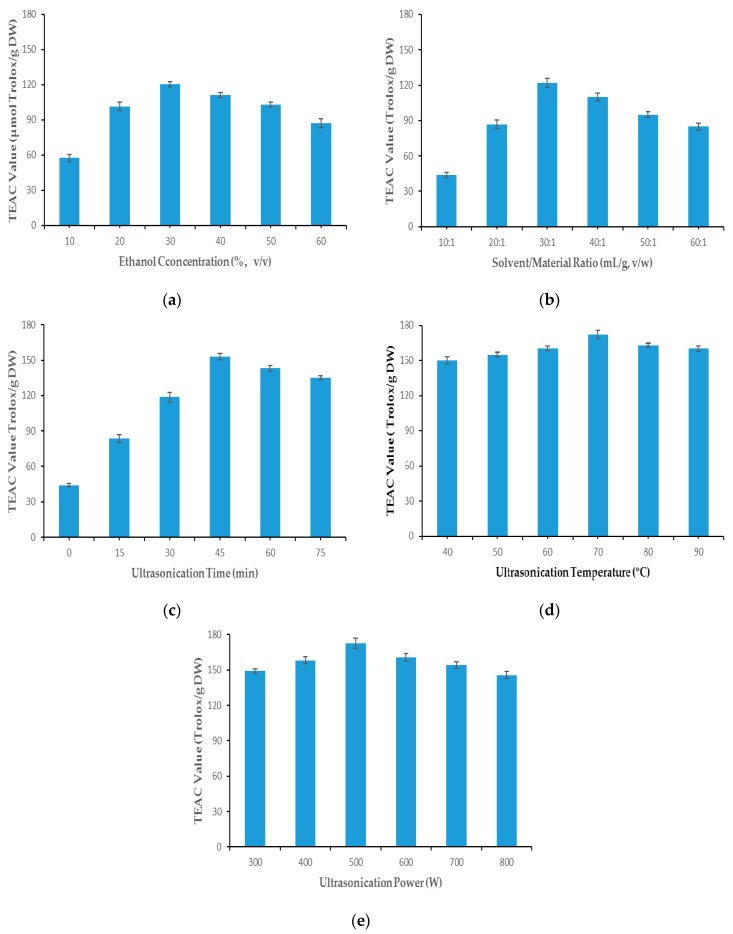
Single-factor experiments results: (**a**) Ethanol concentration; (**b**) Solvent/material ratio; (**c**) Ultrasonication extraction time; (**d**) Ultrasonication extraction temperature; and (**e**) Ultrasonication power.

Several organic solvents are widely used to extract antioxidants from plant matrix, such as methanol, ethanol, and acetone. Among these frequently-used solvents, ethanol aqueous solution is the safest solvent for the environment and people, and is widely employed in the food industry. The efficiency of extraction could be improved if the concentration of ethanol aqueous solution is optimized [21]. In this study, various concentrations of ethanol aqueous solution (10%, 20%, 30%, 40%, 50%, and 60%) were investigated in the condition of S/M ratio 30:1 mL/g, ultrasonication time 30 min, ultrasonication temperature 40 °C and ultrasonication power 500 W. According to the results illustrated in Figure 1a, the antioxidants extracted grew up with the concentration of ethanol increasing from 10% to 30%, reached the peak (120.29 ± 2.31 µmol Trolox/g DW) at 30% ethanol concentration, and then went down dramatically with ethanol concentration increasing. Therefore, 30% ethanol was chosen for the subsequent experiment.

#### 2.1.2. Solvent/Material Ratio

A certain degree of enhancement of S/M ratio might improve efficiency of extraction, which is possible because of a greater concentration difference [22,23]. The influence of the various S/M ratio of (10:1, 20:1, 30:1, 40:1, 50:1 and 60:1 mL/g, *v*/*w*) on the extraction efficiencies of antioxidants from GMBC was investigated with the ethanol concentration of 30%, other parameters were the same as those in the single-factor experiment of ethanol concentration, and the results are displayed in Figure 1b. It is illustrated that the S/M ratio which obtained the highest antioxidant ability (122.68 ± 3.73 µmol Trolox/g DW) was at 30:1 mL/g. The antioxidant ability of the extract grows significantly with S/M ratio from 10:1 to 30:1 and fell gradually with the S/M ratio increasing from 30:1 to 60:1. Thus, the selected S/M ratio for the next step was 30:1.

#### 2.1.3. Ultrasonication Time

The effect of various ultrasonication time (0, 15, 30, 45, 60, and 75 min) on the antioxidant ability of GMBC extraction was investigated with solvent/material ratio 30:1 mL/g, other parameters were the same as those in the single-factor experiment of S/M ratio, and the results are displayed in Figure 1c. While the ultrasonication extracting time grew from 0 min to 45 min, the antioxidant ability of the GMBC extraction rose from 44.25 ± 1.34 µmol Trolox/g DW to 153.73 ± 2.71 µmol Trolox/g DW. If the ultrasonication extraction time continued increasing from 45 min to 75 min, the antioxidant ability of the GMBC extraction showed a decreasing trend. The antioxidant ability increased with the extracting time at first and then decreased after the optimal extraction time, which showed a similar trend to the studies about *Pisum sativum* [24] and *Codonopsis pilosula* [25]. Apparently, the best extraction time for GMBC was 45 min in this study.

#### 2.1.4. Ultrasonication Temperature

Higher ultrasonication temperature could lead to higher diffusion coefficient of the targeted compounds and improve solubility of compounds in the solvent. Thus, the extraction yield might be improved by the increase of temperature [26]. However, excessively high temperature could sometimes decompose bioactive compounds in the extracts, which decreases the yield of antioxidant [27,28]. Therefore, the ultrasonication extraction temperature should be optimized. Effects of ultrasonication temperature (40, 50, 60, 70, 80, and 90 °C) on the yields of antioxidants were evaluated with ultrasonication time 45 min, other parameters were the same as those in the single-factor experiment of ultrasonication time. According to the results shown in Figure 1d, the extraction efficiencies of antioxidants from GMBC increased steadily with the temperature rising from 40 to 70 °C, peaking at 171.56 ± 3.59 µmol Trolox/g DW when the temperature was 70 °C, and then decreased gradually if the temperature grew from 70 °C to 90 °C. Obviously, the best extraction temperature for GMBC was 70 °C.

#### 2.1.5. Ultrasonication Power

The yield of antioxidants in UAE process was also influenced by ultrasonication power. Higher ultrasonication power leads to formation and collapse of more bubbles. Ultrasound waves with a larger amplitude travel through extracting solution so the increase of ultrasonication power might increase the yield of antioxidants [29,30]. However, excessively high ultrasonic power could degrade or decompose the antioxidant ingredients in the extracts, so the optimal ultrasonication power should be investigated. The effects of various ultrasonication powers (300, 400, 500, 600, 700, and 800 W) on the yield of antioxidants from GMBC were evaluated when ultrasonication extracting temperature was 70 °C, other parameters were the same as those in the single-factor experiment of ultrasonication extracting temperature. The results are shown in Figure 1e, the extraction efficiency grew slightly with the increase of ultrasonication power at first, and when the ultrasonication power was 500 W, the antioxidant ability of the extracts was the highest (172.71 ± 4.14 µmol Trolox/g DW). However, the extraction efficiency showed a decreasing trend when the ultrasonication power enhanced from 500 W to 800 W. Thus, 500 W was the best ultrasonication power for extracting antioxidant from GMBC.

### 2.2. Results of Response Surface Methodology Experiment

#### 2.2.1. Central Composite Rotatable Design

In order to optimize the antioxidant ability of the extracts of GMBC, RSM was conducted with a central composite rotatable design (CCRD). In this study, CCRD based on three variables and five levels were generated. Based on the single-factor experiments, three principle factors (concentration of ethanol, ultrasonication extracting time, and solvent/material ratio) were chosen in response surface methodology design. Different levels of solvent/material ratio, ethanol concentration, and ultrasonication extraction time showed significant influence on antioxidant ability of extracts. Twenty experimental runs and the data obtained are illustrated in Table 1. Data suggested that the antioxidant abilities of GMBC were within the range from 93.305 to 178.869 µmol Trolox/g DW.

#### 2.2.2. Fitting Model

A quadratic polynomial model using multiple regression analysis could describe the results of the CCRD. Three independent variables X_1_ (ethanol concentration), X_2_ (S/M ratio), and X_3_ (ultrasonication time) were coded in five levels. The coded levels of X_1_, X_2_, and X_3_ and response variable Y (value of TEAC) were analyzed. ANOVA (analysis of variance) for the fitted equation is illustrated in Table 2. *F* test was conducted to check whether the regression equation was statistically significant. The *F* value was high (41.87) and the *p* value was low (<0.0001), which implied the model obtained was statistically significant. Besides, the determination coefficient value (R^2^) was 0.9741, and the adjusted R^2^ value (Adj. R^2^) was 0.9509, which implied strong correlation between the predicted results and the actual results [31]. In addition, the lack of fit was not significant (*F* = 0.48; *p* = 0.7771 > 0.05), indicating the variation is predicted by the model [32]. These data revealed that the model was appropriate for forecasting TEAC values of GMBC within the tested ranges. In this model, the linear parameters (X_1_, X_2_) were significant and positive at the level of *p* < 0.01, linear parameter X_3_ was positive and statistically significant (*p* < 0.05), quadratic terms (X_1_^2^, X_2_^2^, X_3_^2^) were negative and significant at the level of *p* < 0.01, interaction parameter X_2_X_3_ was negative and significant (*p* < 0.05), whereas interaction terms (X_1_X_2_, X_1_X_3_) were not significant (*p* > 0.05). After discarding the insignificant parameters, the regression model was modified as below:

Y = 170.85 + 14.46X_1_ + 16.86 X_2_ + 4.10X_3_ − 5.08X_2_X_3_ − 9.55X_1_^2^ − 16.25X_2_^2^ − 10.41X_3_^2^ (1)

#### 2.2.3. Effect of Independent Variables on TEAC Value in the RSM Model

**Figure 2:**
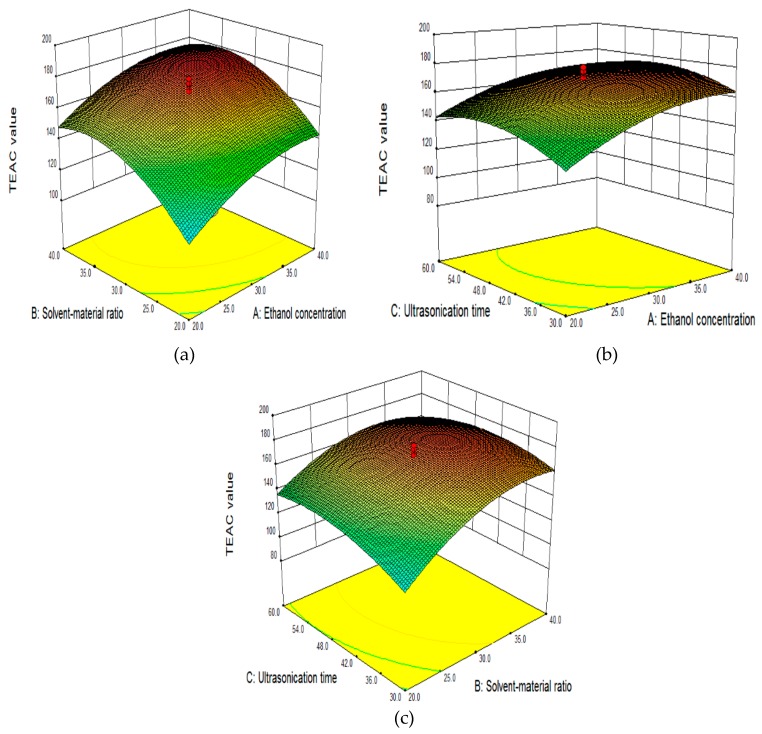
RSM analysis for UAE of antioxidant ingredients from GMBC in relation to concentration of ethanol and S/M ratio (**a**); concentration of ethanol and ultrasonication extracting time (**b**); S/M ratio and ultrasonication extracting time (**c**).

The three-dimensional figures of the response surfaces in Figure 2a–c illustrated the relationship between dependent variable (Y, TEAC value) and independent variables (X_1_, concentration of ethanol; X_2_, S/M ratio and X_3_, ultrasonication extraction time). The response surface showed in Figure 2a was produced according to variations of ethanol concentration and solvent/material ratio, while the ultrasonication extraction time was kept at 35 min. Both the ethanol concentration and the solvent/material ratio showed an influence on the TEAC value. The TEAC values raised gradually at low ethanol concentrations (before around 35% of ethanol concentration) and then decreased slightly when the ethanol concentration increased further, which was possible because solvent polarity changed with the ethanol concentration [19]. The TEAC value showed a similar variation trend when the solvent/material ratio changed. A higher solvent/material ratio might enhance the extraction yield of antioxidant, which is related to a larger difference of concentration between the interior plant cells and the exterior solvent. However, the ultrasonic energy attached to the unit volume would decrease if the solvent/material ratio increased excessively [33,34,35]. As shown in Figure 2b, TEAC value changed with the ethanol concentration or the ultrasonication extraction time if solvent/material ratio was kept as 30:1 mL/g. Ethanol concentration demonstrated a positive effect on the TEAC values. By contrast, the TEAC value increased steadily at first and then fell gradually with the growth of the ultrasonication extraction time. Plant cells are disrupted more by longer extracting time, and the release and diffusion of the antioxidants are enhanced. However, when the extracting time is longer than the optimum, the antioxidants might be degraded [33]. Figure 2c displays the interactive influence of solvent/material ratio and the ultrasonication extraction time when the ethanol concentration was kept at 30% (*v*/*v*). The solvent/material ratio displayed a dramatically positive effect on the TEAC values, which was possible because a longer extracting time might increase ultrasonic effect per unit volume. However, the ultrasonication extraction time showed only a relative limited influence. Based on the results of the response surface plots and ANOVA, it is obvious that ethanol concentration (*p* < 0.01) and solvent/material ratio (*p* < 0.01) were the main parameters influencing the TEAC value, followed by the ultrasonication extraction time (*p* < 0.05).

#### 2.2.4. Verification Experiments and Polyphenolic Compound Profile

The optimum extraction condition was based on analysis of the quadratic polynomial regression model. The best extraction conditions were as follows: 37.6% for ethanol concentration, 35.1:1 mL/g for S/M ratio, and 46.1 min for ultrasonication extraction time. The predicted TEAC value under this condition was 180.75 µmol Trolox/g DW. The predicted condition was applied in verification experiment, and the actual result just matched with the predicted TEAC value (showed in Table 3). As a result, the adequacy and validity of the obtained regression models were confirmed. Besides, the HPLC method was employed to measure the contents of main antioxidants in the GMBC extract [13]. The results showed that the main polyphenols were as follows: vitexin 15.28 ± 1.07 g/kg DW, isovitexin 23.74 ± 1.24 g/kg DW, gallic acid 1.23 ± 0.01 g/kg DW, *p*-coumaric acid 1.80 ± 0.04 g/kg, catechin 1.91 ± 0.12 g/kg, and rutin 0.11 ± 0.01 g/kg DW.

#### 2.2.5. Comparison of UAE with Conventional Methods

The TEAC values were 178.28 ± 7.39, 158.66 ± 4.73, 138.42 ± 3.63 µmol Trolox/g DW for UAE, maceration, and Soxhlet extraction methods, respectively. The total phenolic contents (TPC) values and total flavonoid contents (TFC) values are also showed in Table 4. The data indicated that the antioxidant yield was the highest and the extraction time was the shortest in the UAE process. As a result, UAE was the most effective in three methods. During UAE process, plant cells were destructed by ultrasound cavitation and therefore enhanced the contact of the solvent and the powder, which might be the reason of the high yields of antioxidants in UAE [36]. UAE has shown higher effectiveness in extracting antioxidants from a number of plants, such as pine needles [37] and the flower of *Jatropha integerrima* [38].

## 3. Materials and Methods

### 3.1. Reagents and Sample Preparation

6-Hydroxy-2,5,7,8-tetramethylchromane-2-carboxylic acid (Trolox), 2,2′-azinobis (3-ethylbenothiazoline-6-sulphonic acid) diammonium salt (ABTS), and several antioxidant standards (*p*-coumaric acid, gallic acid, rutin and catechin) were purchased from Sigma-Aldrich (St. Louis, MO, USA). Other antioxidant standards (vitexin, isovitexin) were from Biopurify Phytochemicals (Chengdu, China). Potassium persulfate was produced by Tianjin Chemical Factory (Tianjin, China). Ethanol and methanol were purchased from Kelong Chemical Factory (Chengdu, China). All reagents applied were of analytical grade in this study.

The coats of mung bean (63.5% moisture) were separated from the sprouts, cleaned with deionized water, dried at 35 °C, and then were ground into fine GMBC powders (1.8% residual moisture) by a special pulverizer (model XT-A400, Hongtaiyang Co., Ltd., Yongkang, Zhejiang, China). The GMBC powder was stored at 4 °C in the refrigerator before use.

### 3.2. Extraction Section

#### 3.2.1. UAE Procedure

The UAE procedure was conducted based on the method reported by Li et al., and a few changes were made [19]. The powder of GMBC (0.10 g) was put into a centrifuge tube (15 mL), mixed with ethanol aqueous solution (the volume and the ethanol concentration were according to the study design). The tube containing the mixture was set in ultrasonic water bath device (model Kj1012B, Kejin Co., Ltd., Guangzhou, China) under the designed conditions. After ultrasonic extraction, the samples were centrifuged (5000× *g* for 25 min, and then the epipelagic solution was gathered for the step of measurement of TEAC value. Besides, for the high-performance liquid chromatography analysis, the solution was filtered using 0.20 μm membrane (Merck Millipore, Cork, Ireland).

#### 3.2.2. Conventional Methods

Maceration extraction: The powder of GMBC (0.10 g) was put in a centrifuge tube (15 mL), added with 3.51 mL of 37.6% ethanol, and extracted for 24 h at room temperature (25 °C). After the extraction, the mixture was centrifuged for 25 min at 5000× *g*, and then the epipelagic solution was gathered for determining TEAC value.

Soxhlet extraction: The powder of the GMBC (1.0 g) was wrapped with Whatman filter paper, and was put into a Soxhlet extractor with 350 mL of 37.6% ethanol aqueous solution. The extraction procedure was conducted at 95 °C for 4 h. The obtained solution of extraction was cooled to room temperature and then collected for determining TEAC value.

### 3.3. Determination of the Yield of Antioxidants

Trolox equivalent antioxidant capacity (TEAC value): TEAC assay is commonly used in determination of antioxidant ability in recent years, for it is efficient and sensitive, and presents a global measure of antioxidant ability with a wide linear reaction range [39]. In this paper, TEAC assay was applied to evaluate antioxidant ability of GMBC extracts, and was performed based on the method in the published literature [19]. To prepare stock solution of ABTS^•+^, 5.0 mL ABTS solution (7 mmol/L), and 5.0 mL potassium persulfate (2.45 mmol/L) were mixed and put into a capped tube and stored in the dark for 16 h. Then the ABTS^•+^ stock solution was obtained, and it should be used within 48 h. The prepared stock solution of ABTS^•+^ was diluted, and the working solution of ABTS^•+^ was obtained when the absorbance reached 0.70 ± 0.05 at λ_734 nm_. Then, 3.8 mL working solution of ABTS^•+^ and 100 µL diluted sample were mixed and incubated for 6 min. After that, ultraviolet spectrometry was used to determine the absorbance of the mixed solution at λ_734 nm_. The antioxidant activity was presented as Trolox equivalent as Trolox was applied as a reference standard. The unit of TEAC value was µmol Trolox/g DW (dry weight). The ABTS^•+^ stock solution and ABTS^•+^ working solution should not be exposed to light until be used.

Total phenolic contents (TPC value): TPC value of GMBC extract was investigated according to the published method [7]. Gallic acid was used as the reference standard, and the TPC value was displayed as mg gallic acid equivalent (GAE)/g.

Total flavonoid contents (TFC value): TFC of GMBC extract was investigated according to the literature of Kim et al. [40]. Catechin was chosen as standard of reference in this study, and the outcome of total flavonoid contents was displayed as mg catechin equivalent (mg CE)/g.

### 3.4. High-Performance Liquid Chromatography Analysis of Antioxidant Components

The antioxidant components of the GMBC extract obtained under the optimal condition were investigated by high-performance liquid chromatography (HPLC) according to our previous method [12]. In brief, analysis of HPLC was conducted with a Prominence Modular HPLC system, which is made up of a binary pump, an online degasser, an auto-sampler, and a photodiode array detector. An Agilent Zorbax SBC18 column (4.6 mm × 150 mm, 3.5 µm) (Agilent Technologies, Santa Clara, CA, USA) was employed to separate antioxidant ingredients from the extracts. The mobile phase was made up of solution A (2.5% formic acid aqueous solution) and solution B (100% methanol). The gradient elution (the flow rate, 0.8 mL/min; the injection volume, 20 µL) was performed as follows: 0–15 min, 5%–30% B; 15–40 min, 30%–40% B; 40–45 min, 40%–50% B; 45–50 min, 50%–95% B; 50–60 min, 95% B; 60–65 min, 95%–5% B; 65–75 min, 5% B. Catechin and gallic acid were detected at 280 nm, *p*-coumaric acid, vitexin, and isovitexin were detected at 320 nm, and rutin was detected at 350 nm. The retention time and spectra of antioxidants were compared with the standard compounds, and were quantified according to the peak areas. The result was showed as g/kg DW (dry weight) of GMBC.

### 3.5. Design of the Experiment

#### 3.5.1. Single-Factor Experiments

In order to investigate the influence of every factor on TEAC value of the GMBC extract, single-factor experiments were conducted for determining the effect of five different factors. Concentration of ethanol (10%–60%), S/M ratio (10:1–60:1 mL/g), ultrasonication extracting time (0–75 min), ultrasonication temperature (40–90 °C), and ultrasonication power (300–800 W) were determined on the basis of the yield of antioxidants.

#### 3.5.2. Response Surface Methodology

A RSM with three-factor, five-level CCRD was conducted to optimize the antioxidant ability of the GMBC extracts. Based on the outcome of the single-factor experiments, three main factors were selected for response surface design. The appropriate conditions were 30% of ethanol concentration, 30:1 of S/M ratio and 45 min of ultrasonication time, and they were chosen as the central condition of CCRD (showed in Table 5). The 20 experimental runs which include the central point (six replicates) were performed (Table 1). A quadratic polynomial model using multiple regression analysis could express the results obtained in RSM. The regression model performed is as below:

Y = β_0_ + ∑β_i_X_i_ + ∑β_ii_X_i_^2^ + ∑β_ij_X_i_X_j_ (2)

In the second-order polynomial model, Y stands for the response value (dependent variable), and X_i_ and X_j_ stand for the independent variables. For the regression coefficients, β_i_ is linear coefficient, β_ij_ is quadratic coefficient, β_ii_ is interaction coefficient, and β_0_ is a constant.

### 3.6. Statistical Analysis

Design Expert 8.06.1 was applied in statistical analysis of results in RSM experiment. SPSS 19.0 and Microsoft Excel 2016 were used in statistical analysis during all the study. Multiple regression analysis and ANOVA were performed by Design Expert 8.06.1 (Stat-Ease, Minneapolis, MN, USA) and *p* value, *F* value, lack-of fit test, R^2^, and Adj. R^2^ were obtained to evaluate the models.

## 4. Conclusions

In this study, a UAE method by RSM optimization has been developed to extract antioxidants from GMBC. The obtained regression model showed high correlation (R^2^ = 0.9741, Adj. R^2^ = 0.9509) which suggested that it could precisely show the effect of the three main factors on the TEAC value. The optimal extraction condition according to model was ethanol aqueous solution concentration of 37.6%, S/M ratio of 35.1:1 mL/g, ultrasonication time of 46.1 min, ultrasonication temperature of 70 °C, and ultrasonication power of 500 W. The predicted antioxidant capacity under this condition was 180.75 µmol Trolox/g DW. The actual results (178.28 ± 7.39 µmol Trolox/g DW) matched with predicted values in the verification experiment. Compared with conventional methods (maceration extraction method and Soxhlet extraction method), optimized UAE was much more efficient for extracting antioxidant ingredients from GMBC. Besides, the main antioxidant components in GMBC extract are catechin, gallic acid, *p*-coumaric acid, vitexin, and isovitexin. GMBC is a great source of antioxidants for its low cost, large production, and high content of polyphenolic compounds. The GMBC extract could be used as a food additive for preserving food that can be susceptible to oxidization or pharmaceuticals for the prevention and treatment of oxidative stress-induced diseases.

## Figures and Tables

**Table 1:** Twenty experimental runs of RSM analysis and the corresponding experimental results.

Run	X_1_ (Ethanol Concentration, %, *v*/*v*)	X_2_ (Solvent/Material Ratio, mL/g)	X_3_ (Ultrasonication Extraction Time, min)	Response Y (TEAC Value, µmol Trolox/g DW)
1	30	13.2	45	96.917
2	20	20	60	115.408
3 *	30	30	45	168.774
4	30	30	70.2	146.164
5	20	40	60	143.642
6 *	30	30	45	159.208
7 *	30	30	45	170.878
8	30	30	19.8	132.018
9	20	20	30	93.305
10	40	20	60	139.380
11	13.2	30	45	114.183
12	40	40	60	162.812
13	30	46.8	45	148.213
14	46.8	30	45	168.825
15	40	40	30	170.658
16 *	30	30	45	172.434
17	40	20	30	125.065
18 *	30	30	45	175.756
19 *	30	30	45	178.869
20	20	40	30	140.013

* Six replicates of central point.

**Table 2:** Analysis of variance (ANOVA) for the response surface model.

Source	Sum of Squares	df	Mean Square	*F* Value	*p* Value	Significant
Model	12903.46	9	1433.72	41.87	< 0.0001	significant
X_1_	2854.53	1	2854.53	83.36	< 0.0001	
X_2_	3881.47	1	3881.47	113.35	< 0.0001	
X_3_	229.56	1	229.56	6.70	0.0270	
X_1_X_2_	4.38	1	4.38	0.13	0.7282	
X_1_X_3_	46.38	1	46.38	1.35	0.2715	
X_2_X_3_	206.40	1	206.40	6.03	0.0340	
X_1_^2^	1315.57	1	1315.57	38.42	0.0001	
X_2_^2^	3805.67	1	3805.67	111.13	< 0.0001	
X_3_^2^	1561.00	1	1561.00	45.58	< 0.0001	
Residual	342.45	10	34.24			
Lack of fit	111.83	5	22.37	0.48	0.7771	not significant
Pure error	230.62	5	46.12			
Cor total	13245.91	19				
R^2^ = 0.9741						
Adj. R^2^ = 0.9509						

**Table 3:** Verification experiments.

Optimal Condition			TEAC Value (µmol Trolox/g DW)	
Ethanol Concentration	Solvent/Material Ratio	Extraction Time	Experimental Result	Predicted Value
37.6%	35.1 mL/g	46.1 min	178.28 ± 7.39	180.75

**Table 4:** Comparison of UAE with conventional methods.

Extraction Method	Ethanol Concentration (%)	Extraction Temperature (°C)	Extraction Time	TEAC Value (µmol Trolox/g DW)	TPC Value (mg GAE/g DW)	TFC (mg CE/g DW)
UAE	37.6%	70	46.1 min	178.28 ± 7.39	33.91 ± 1.06	15.06 ± 1.11
Maceration	37.6%	25	24 h	158.66 ± 4.73	23.64 ± 1.28	6.67 ± 0.26
Soxhlet	37.6%	95	4 h	138.42 ± 3.63	19.96 ± 1.37	4.02 ± 0.18

**Table 5:** The levels of the main factors of the extraction process.

Independent Variables	Coded Levels				
	−1.68	−1	0	1	1.68
X_1_ (ethanol concentration, %, *v*/*v*)	13.2	20	30	40	46.8
X_2_ (solvent/material ratio, mL/g)	13.2	20	30	40	46.8
X_3_ (ultrasonication time, min)	19.8	30	45	60	70.2
